# 

**Published:** 1959-07

**Authors:** 


### THE MEDICAL JOURNAL OF THE SOUTH-WEST

*Incorporating*
*The Bristol Medico-Chirurgical Journal*

NATIONAL LIBRARY
NOV 10 1959
OF MEDICINE

*July 1959*

VOL. 74 (iii). No. 273

